# A Platform to View Huntingtin Exon 1 Aggregation Flux in the Cell Reveals Divergent Influences from Chaperones hsp40 and hsp70*

**DOI:** 10.1074/jbc.M113.486944

**Published:** 2013-11-06

**Authors:** Angelique R. Ormsby, Yasmin M. Ramdzan, Yee-Foong Mok, Kristijan D. Jovanoski, Danny M. Hatters

**Affiliations:** From the Department of Biochemistry and Molecular Biology, Bio21 Molecular Science and Biotechnology Institute, 30 Flemington Road, University of Melbourne, Melbourne, Victoria 3010, Australia

**Keywords:** Amyloid, Analytical Ultracentrifugation, Chaperone Chaperonin, Flow Cytometry, Protein Aggregation, PulSA, huntingtin, Oligomers, Polyglutamine

## Abstract

Our capacity for tracking how misfolded proteins aggregate inside a cell and how different aggregation states impact cell biology remains enigmatic. To address this, we built a new toolkit that enabled the high throughput tracking of individual cells enriched with polyglutamine-expanded Htt exon 1 (Httex1) monomers, oligomers, and inclusions using biosensors of aggregation state and flow cytometry pulse shape analysis. Supplemented with gel filtration chromatography and fluorescence-adapted sedimentation velocity analysis of cell lysates, we collated a multidimensional view of Httex1 aggregation in cells with respect to time, polyglutamine length, expression levels, cell survival, and overexpression of protein quality control chaperones hsp40 (DNAJB1) and hsp70 (HSPA1A). Cell death rates trended higher for Neuro2a cells containing Httex1 in inclusions than with Httex1 dispersed through the cytosol at time points of expression over 2 days. hsp40 stabilized monomers and suppressed inclusion formation but did not otherwise change Httex1 toxicity. hsp70, however, had no major effect on aggregation of Httex1 but increased the survival rate of cells with inclusions. hsp40 and hsp70 also increased levels of a second bicistronic reporter of Httex1 expression, mKate2, and increased total numbers of cells in culture, suggesting these chaperones partly rectify Httex1-induced deficiencies in quality control and growth rates. Collectively, these data suggest that Httex1 overstretches the protein quality control resources and that the defects can be partly rescued by overexpression of hsp40 and hsp70. Importantly, these effects occurred in a pronounced manner for soluble Httex1, which points to Httex1 aggregation occurring subsequently to more acute impacts on the cell.

## Teaser

**Background:** How misfolded proteins such as mutant huntingtin aggregate in the cell remains enigmatic.

**Results:** We built a platform to view how aggregation proceeds and assessed the impact of quality control chaperones hsp40 and hsp70.

**Conclusion:** hsp70 enhanced survival of cells with aggregates; hsp40 suppressed aggregation.

**Significance:** We developed a new toolkit to illustrate the impact of protein aggregation on cell biology.

## Introduction

Protein misfolding and aggregation into β-sheet rich amyloid fibrils is a hallmark and possible cause of at least 36 human diseases (1). Our basic knowledge of protein misfolding and how the inherent features of proteins and physicochemical environment influence aggregation has largely come from purified peptide and protein model systems (2–5). A common theme has emerged that aggregates, notably small oligomeric forms, can incur toxicity to cells (6, 7). When aggregates form in a cell, however, they do so in an environment of omnifarious influence from protein quality control mechanisms (8). Protein quality control processes change the landscape by which proteins spontaneously aggregate by refolding misfolded conformations, disaggregating aggregates, degrading them, and sorting them into different cellular locations such as aggresomes, insoluble protein deposits, and Juxta nuclear quality control compartments for deposition and sequestration (9–14).

The active movement of misfolded proteins into deposits such as aggresomes presents a paradox to the study of the effect of protein misfolding and aggregation *in situ*. On the one hand, organized aggregation by protein quality control machinery has net benefits to cell health, and on the other hand, spontaneous inappropriate aggregation is capricious (15). It remains plausible that both phenomena occur at the same time especially when protein quality control loses its capacity to control aggregation in an organized fashion (16). The contradictory nature of these processes and their relevance to disease necessitates more sophisticated approaches to decipher the molecular process of aggregation inside the cell and how this may impact cell health (15).

With this in mind, we developed new toolkits to more precisely probe the intracellular conformation and aggregation state of the exon 1 fragment of mutant huntingtin protein (Httex1)^2^ in cells, which has a number of attractive features for this problem (17–19). Mutant Httex1 accumulates as intracellular inclusion bodies in Huntington disease, which is caused by mutations that result in an expansion of a polyglutamine (polyQ) sequence within Httex1 to beyond a threshold of 36 glutamines (20, 21). PolyQ expansions in the pathological range lead to Httex1 spontaneously assembling into amyloid-like fibrils in a manner that is faster for longer polyQ lengths (22, 23). Expression of polyQ-expanded proteins in animals and cells recapitulates aggregation and pathology in a polyQ length-dependent manner, demonstrating clear links between the intrinsic biophysical attributes of Httex1, aggregation, and pathology (24–26).

Our first toolkit involved the development of tetracysteine-based biosensors for detecting the earliest oligomerization steps of the Httex1 in live cells (17). The TC9 sensor is a derivative of Httex1 with an engineered tetracysteine (TC) tag embedded with the Httex1 sequence that is masked from binding to biarsenical fluorescent dyes upon self-assembly *in vitro* (17). Httex1^TC9^ is also tagged C-terminally with a fluorescent protein (*e.g.* cyan fluorescent protein derivative Cerulean) that independently reports the presence of the protein. Hence, two-color imaging enables readouts of the balance of monomers and aggregates inside live cells, independently to cellular localization (17). This technology was recently merged with a flow cytometry pulse shape analysis (PulSA) method, which utilizes fluorescent pulse width and height information from a flow cytometer to monitor changes in the intracellular distribution of protein (19). PulSA in combination with the TC9 sensor system enabled a distinction in detection of biochemical aggregates, which can be as small in theory as a dimer (*i.e.* nanometer scale), from the condensation into microscopically visible structures (*i.e.* micrometer scale) such as inclusions, providing a new high throughput capacity to track cells enriched with dispersed oligomers of Httex1 from cells with monomers or the inclusions.

A second toolkit was sedimentation velocity analysis (SVA) with analytical ultracentrifugation to quantitate the oligomeric size and heterogeneity of GFP-tagged Httex1 aggregate forms in a cell lysate (18). For the aggregation prone 46Gln form of Httex1, this approach yielded a heterogeneous mixture of oligomers, most abundantly about 30 nm in diameter. The nonaggregation 25Gln isoform of Httex1 in contrast only yielded monomers. The combination of the single cell approaches with biochemical approaches (*e.g.* SVA) in principle provides an enabling platform to define the kinetic process of aggregation approaching a molecular scale of detail.

Here, we describe an implementation of an integrated platform for defining Httex1 aggregation in the cell by merging our existing toolkits together and developing new capabilities to follow cell death and protein levels. We used this workflow to first monitor the impact of aggregation state on cell death, and second to examine how elevation of key inducible members of the heat shock protein family (hsp70 protein HSPA1A and its hsp40 cofactor DNAJB1) alters the Httex1 aggregation landscape and cell survival when levels are elevated. hsp70 and its co-chaperone hsp40 are key elements that have canonical functions in assisting proteins to fold correctly, and they potently inhibit toxicity of Httex1 in model systems (27–30). How they do this remains enigmatic because protection does not always occur with reducing inclusions, which seems counterintuitive to their canonical role in assisting proteins to fold (30–36).

## EXPERIMENTAL PROCEDURES

### 

#### 

##### Cloning of Constructs

The TC9 variant of Httex1 was generated as described (17). The Httex1-Emerald constructs were produced as described (18) to produce Httex1 with a C-terminal Emerald fusion in the pT-Rex vector backbone (Invitrogen).

The IRES vectors were made by inserting an IRES sequence C-terminally to the Httex1^TC9^-Cerulean moiety in the pT-Rex backbone. Specifically, we ligated the following synthetic gene (Geneart, Invitrogen) cut from the cloning vector with MfeI and EcoRI into a unique EcoRI site at the 3′ of the stop codon of Httex1^TC9^-Cerulean (Sequence 1), where key features are annotated as follows: CAATTG, MfeI restriction site; ***SEQUENCE***, IRES sequence; SEQUENCE, mKate2; shaded SEQUENCE (37), farnesylation tag; CGTACG with dots below, BsiW1 restriction site; GAATTC with rule and dots below, EcoRI restriction site. The nonfarnesylated mKate2 version of the IRES vector was created by excision of the farnesylation tag with BsiW1 digestion and vector religation. Hsp40 and Hsp70 chaperones were provided from Paul Muchowski (Gladstone Institutes) and verified by DNA sequencing.

**SEQUENCE 1:**
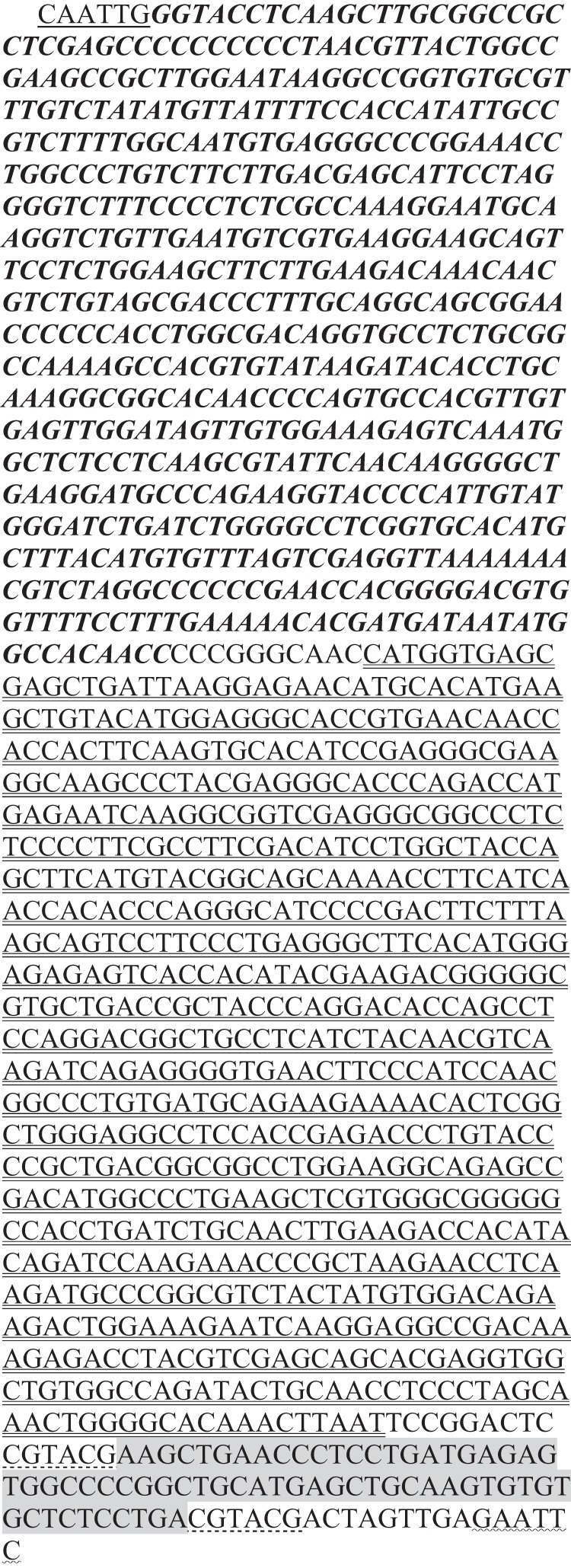


##### General Cell Culture

Neuro-2a cells were maintained in OptiMEM (Invitrogen) supplemented with 10% fetal calf serum, 1 mm glutamine, 100 units/ml penicillin, and 100 μg/ml streptomycin in a humidified incubator with 5% atmospheric CO_2_.

##### Cell Preparation for Flow Cytometry

2 × 10^5^ cells were plated in individual wells of a 24-well tissue culture plate. The following day, the cells in each well were transfected with 2 μl of Lipofectamine 2000/0.8 μg of vector DNA according to the manufacturer's instructions (Invitrogen). The next day, the media were refreshed (500 μl), and for the time course the media were refreshed daily. Cell suspensions were kept on ice until analysis by flow cytometry.

FlAsH staining was performed as described previously (19) with the exception of using a 12-well tissue culture plate setup, and all volumes used during the transfection and FlAsH staining protocols were doubled.

For SYTOX staining, the media from the cultured cells were removed 24 h after transfection and kept aside in parallel format in 24-well plates and retained in the cell culture incubator. Fresh media were added to the cells, and at further time points of analysis, the media were collected and added to the initial media collections (so as to collect any detached cells). Remaining adherent cells were detached by gentle agitation and pipetting in 500 μl of phosphate-buffered saline (PBS). The cell suspension was added to the set-aside media and then pelleted (1600 × *g*; 3 min; room temperature). The supernatant was discarded and the pellet resuspended in 500 μl of PBS followed by 0.5 μl of 5 μm SYTOX Red Dead stain (Invitrogen). Cell suspensions were kept on ice until analysis by flow cytometry (which was all completed within 1 h after labeling).

##### Flow Cytometry

Cells were analyzed at high flow rate in an LSRFortessa flow cytometer, equipped with 405- and 488-nm lasers (BD Biosciences). 50,000–100,000 events were collected, using a forward scatter threshold of 5,000. Data were collected in pulse height, area, and width parameters for each channel. For Cerulean fluorescence, data were collected with the 405-nm laser and Pacific blue filter. For FlAsH and GFP, data were collected with the 488-nm laser and FITC filter. mKate2 fluorescence was collected in the PE-Texas Red filter. SYTOX Red Dead stain was collected using 640-nm laser and the APC filter. All flow cytometry data were analyzed with FACSDiva software (BD Biosciences), FlowJo (Tree Star Inc.), or manually in Excel (Microsoft).

##### Cell Preparation for Sorting and Imaging

1 × 10^6^ cells were plated in individual wells of a 6-well tissue culture plate. The following day, the cells in each well were transfected with 10 μl of Lipofectamine 2000 and 4 μg of vector DNA according to the manufacturer's instructions (Invitrogen). After 24 h, the media were refreshed (2 ml). Cells were harvested at 48 h post-transfection by first rinsing in PBS, followed by resuspension in PBS with a cell scraper, and gentle pipetting. Cells were pelleted (1,600 × *g*; 3 min) and resuspended in 2 ml of 2% (v/v) paraformaldehyde for 30 min at room temperature. Cells were again pelleted (1,600 × *g*; 3 min), resuspended in 2 ml of PBS, and filtered through 100-μm nylon mesh before analysis and recovery on a BD FacsAria cell sorter (BD Biosciences). Cells were imaged on a Leica SP2 confocal microscope using a HC PL APO lbd.BL 20.0 × 0.70 IMM objective (TCS SP2 Leica).

##### Gel Filtration Chromatography

6 × 10^6^ cells were plated in 75-cm^2^ tissue culture flasks. The following day, cells were transfected with 24 μg of DNA and 60 μl of Lipofectamine 2000. 24 h after transfection, media were either refreshed for the 30-h time point, or cells were harvested by scraping. Cells were then pelleted (1,600 × *g*; 3 min; room temperature) and snap-frozen in liquid nitrogen. Cells were then lysed as described previously (18).

Running buffer (20 mm Tris, pH 7.4, 150 mm NaCl, 1% Triton X-100) was used to pack and equilibrate Sephacryl S-1000 superfine medium (GE Healthcare) into a 1.0 × 30-cm chromatography Econo-Column (Bio-Rad). 200 μl of cell lysate was run through the column at a flow rate of 1.1 ml/min. 4-Drop fractions were collected into three U-shaped black-bottomed 96-well plates. Fluorescence of fractions was assessed with a Varioskan Flash spectral scanning multimode plate reader. Excitation/emission wavelength of 470/511 nm was used.

##### SVA

Cell preparation, lysis, and analysis were all performed as described previously (18).

##### Statistical Analysis

Data were analyzed for differences by either a one- or three-way ANOVA with the Holm-Sidak Test comparing each chaperone treatment with the Htt alone control.

## RESULTS

**FIGURE 1.**
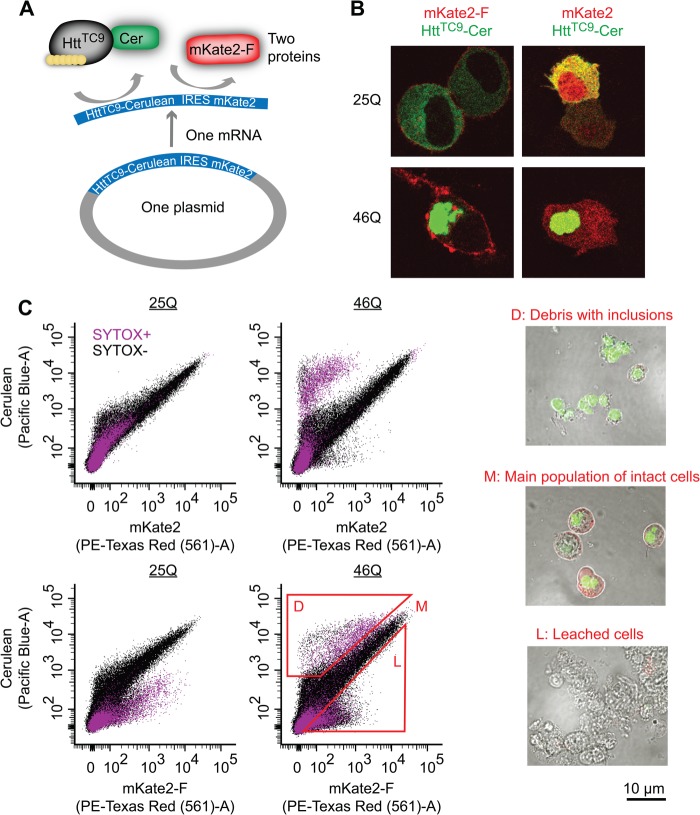
**Biosensor platform to track Httex1 aggregation inside the cells and effect of aggregation on cell viability.** *A,* schematic of the IRES vector system employed. The vector expresses Httex1^TC9^, which is a biosensor of the Httex1 aggregation state that we previously showed can bind to the biarsenical dyes FlAsH or ReAsH when Httex1 is monomeric but not when it aggregates (17). Httex1^TC9^ is fused to Cerulean as a marker to track Httex1 expression levels. Downstream the IRES sequence is the mKate2 fluorescent protein. We created two IRES versions, one with a farnesylation targeting sequence appended to the C terminus of mKate2 (*mKate2-F*) and another lacking this sequence (*mKate2*). *B,* expression and imaging of the IRES constructs in Neuro2a cells with derivatives of Httex1^TC9^ containing different polyQ lengths. *C,* flow cytograms of Neuro2a cells transfected with the IRES vectors. Cells were pre-gated as indicated in Fig. 2. The gates L, M, and D were used for sorting, recovery, and visual inspection by microscopic imaging.

**FIGURE 2.**
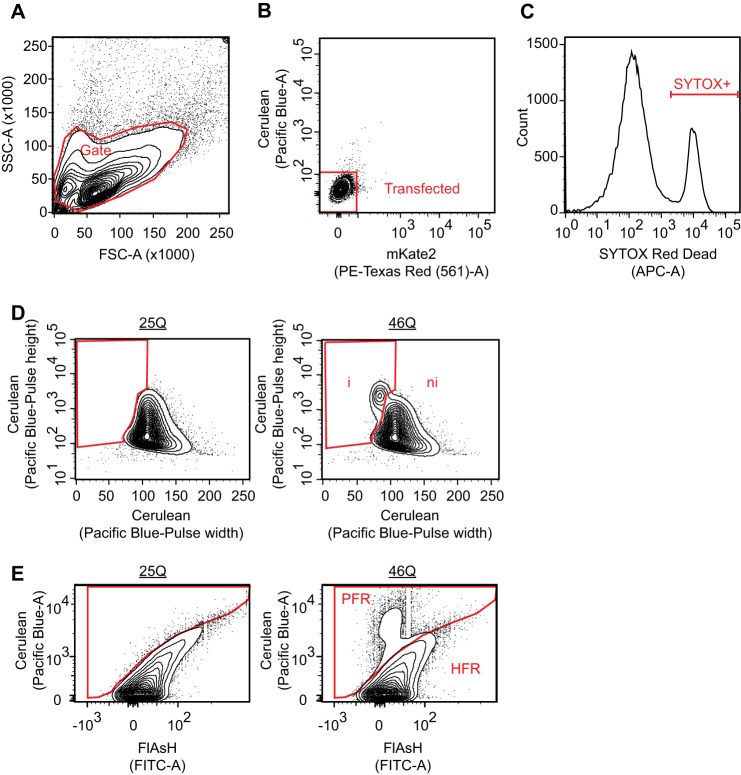
**Gating strategies for the flow cytometry.** *A,* for all analyses in this study, cells were gated to exclude the smallest particles and clumped cells. *B,* untransfected cells were excluded from the analysis using untransfected cells to reference the gate boundary. *C,* SYTOX Red Dead was gated based on permeability into cells. *D,* PuLSA gating strategy of Httex1^TC9^-Cerulean was described previously (19). *E,* FlAsH reactivity of cells expressing Httex1^TC9^-Cerulean was gated into PFR population and high FlAsH-reactive population as described previously (19).

We first developed strategies to examine how the transition of mutant Httex1 from monomers to diffuse oligomers and large inclusions in individual mammalian cells correlate with cell death. Our TC9-based biosensor system of the Htt aggregation state (17) was adapted into a bicistronic expression system (homemade pTIREX vectors) to independently mark cells that had expressed Httex1 with a second membrane-associated red fluorescent protein mKate2 (Fig. 1*A*). The mKate2 was C-terminally tagged with a farnesylation targeting sequence (mKate2-F), which targets proteins to the plasma membrane (39, 40). This was designed to fluorescently detect cells that may have leached their cytosolic contents upon cell death, such as when small diffusible oligomers form, which would render them undetected if we tracked Httex1-Cerulean only (11, 41). mKate2 was anchored to the plasma membrane only when appended with a C-terminal farnesylation tag and was not colocalized with Httex1-Cerulean inclusions (Fig. 1*B*). Cells, including all those detached from the plate, were collected and analyzed by flow cytometry (gating strategy shown in Fig. 2, *A* and *B*). Expression of both pTIREX mKate2 and mKate2-F variants resulted in a “Major” population (M population) displaying a linear correlation between Cerulean and mKate2 fluorescence (Fig. 1*C*), which was absent in an untransfected control (Fig. 2*B*). Recovery and imaging of the M population revealed them to comprise intact cells with diffuse or inclusion-localized Httex1 (Fig. 1*C*). Both pTIREX mKate2 and mKate2-F constructs that contained the aggregation prone 46Gln variant of Htt^TC9^-Cerulean also had a unique small population (D population) with greater Cerulean fluorescence than mKate with respect to the M population. Recovery and imaging confirmed the D population was largely free-floating inclusions attached with cellular debris (Fig. 1*C*). The mKate2-F pTIREX construct had an additional population (L population) enriched with mKate2-F and deficient in Cerulean, which we anticipated for cells that had leached cytosol due to death or a compromised membrane integrity and which was confirmed when these cells were recovered and imaged (Fig. 1*C*). Simultaneous analysis with the dead cell marker SYTOX Red Dead (gating strategy shown in Fig. 2*C*), which binds to nucleic acids in cells with compromised membranes, revealed many species in the D and L populations to be nonreactive to SYTOX, presumably due to a loss of nucleic acids through leaching (Fig. 1*C*). To more fully estimate the number of dead cells, we hence took a strategy of summing cells with SYTOX− reactivity (SYTOX+), additional leached cells (SYTOX−,L), and debris (SYTOX−,D), whereby we assumed each D population member reflected the remnants from one cell.

**FIGURE 3.**
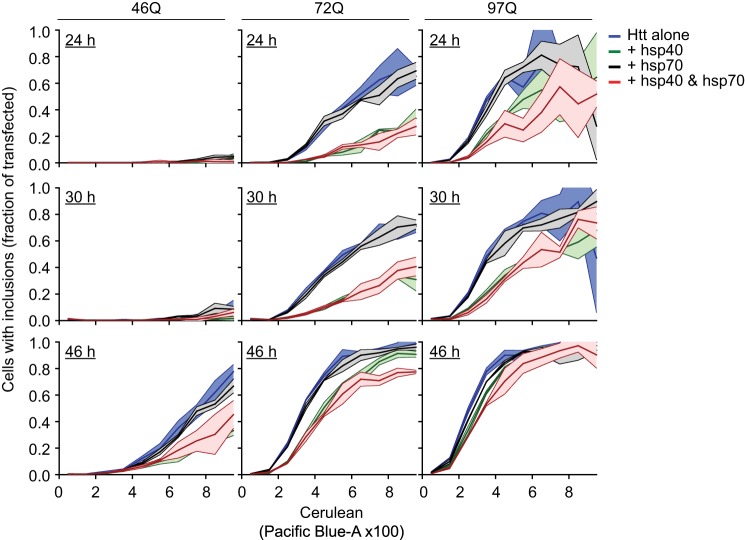
**Aggregation “kinetics” of Httex1 in cells and effect of overexpressing hsp40 and hsp70.** Flow cytometry data of Neuro2a cells transfected with Httex1^TC9^-Cerulean in the mKate2-F IRES vector (pTIREX) is binned into Htt expression level and shown as proportion of cells with inclusions (i population) from total number of cells in each bin. The i population was calculated by gating the pulse width and height values of Cerulean (Pacific-Blue channel) as described previously (19). The Httex1^TC9^-Cerulean IRES vector was coexpressed with a nonfluorescent Y66L mutant of GFP (GFP^inv^) as a “blank” (alone) or with DNAJB1 plus GFP^inv^ (Hsp40), HSPA1A plus GFP^inv^ (Hsp70), or DNAJB1 plus HSPA1A (Hsp40 and Hsp70). GFP^inv^ was used to buffer DNA levels so to enable the same quanta of DNA of Httex1^TC9^-Cerulean (1/3 quantum), and each chaperone (1/3 quantum) was used in transfections for each treatment. Data shows mean (central lines) ± S.D. (outer boundaries); *n* = 3.

We next investigated the influence of up-modulating hsp40 and hsp70 on our aggregation platform, which have previously been shown to alter the Httex1 aggregation process and toxicity (29, 31, 33, 36, 42). With the mKate2-F pTIREX vectors and PulSA, we monitored the rate at which cells expressing Httex1^TC9^-Cerulean of different polyQ lengths formed inclusions when coexpressed with heat shock protein hsp40 family member DNAJB1 and hsp70 member HSPA1A. PulSA enabled cells with inclusions (i population) to be separated from cells with dispersed cytosolic Htt (ni population; gating strategy in Fig. 2*D*) (19). Cells were analyzed by PulSA and divided into bins of expression level (based on Cerulean fluorescence intensity) to define the expression level dependence on inclusion formation and influence from the chaperone treatments (Fig. 3). Essentially, cells formed inclusions in a concentration- and time-dependent manner, which was enhanced with longer pathogenic polyQ lengths. Coexpression of hsp70 had minor, if any, effects on inclusion formation for most time points and polyQ lengths at the different expression levels (Fig. 3). hsp40, by contrast, profoundly suppressed inclusion formation under most time points and polyQ lengths, and coexpression of both chaperones led to no major additional reduction in rates of inclusion formation (Fig. 3).

**FIGURE 4.**
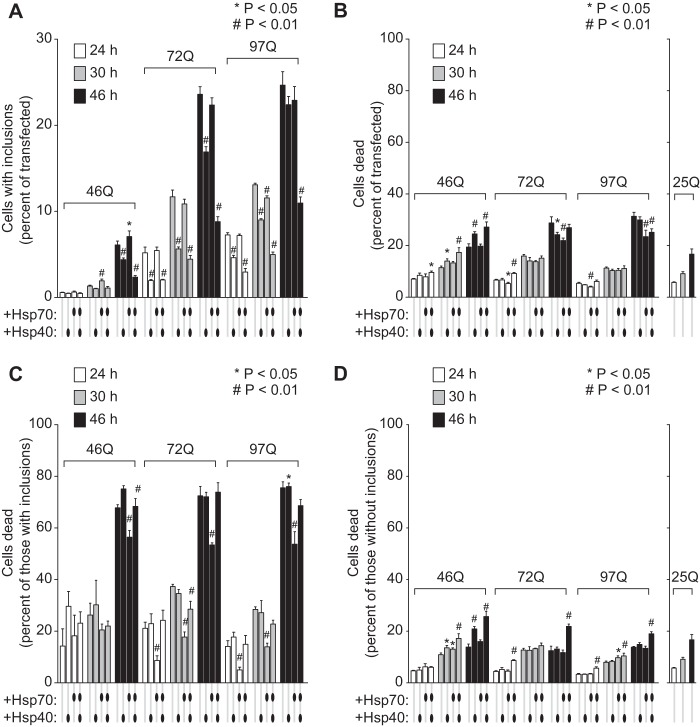
**hsp40 and hsp70 dictate divergent outcomes to inclusion formation rates and toxicity.** *A,* flow cytometry data of all cells expressing the Httex1^TC9^-Cerulean from the IRES mKate2-F vector as proportion of inclusions (i population). Different variants of this construct with 46Gln, 72Gln, and 97Gln are shown and for cells collected at different time points post-transfection. The cotransfections were performed as described in Fig. 3 legend (*n* = 3, mean ± S.D. shown). Three-way ANOVA for each chaperone treatment against Htt alone for all Gln length and time points are as follows: hsp40 (*p* < 0.001), hsp70 (*p* = 0.014), and hsp40 and hsp70 (*p* < 0.001). Individual pairwise comparisons against Htt alone as control are shown for *p* < 0.05. *B,* death rate for the cells. Death rates were determined as the number of free inclusions (D population), leached cells (L population), and further cells reactive to SYTOX Dead Red (*n* = 3, mean ± S.D. shown). Three-way ANOVA for each chaperone treatment against Htt alone for all Gln length and time points are as follows: hsp40 (*p* = 0.949), hsp70 (*p* < 0.001), and hsp40 and hsp70 (*p* < 0.001). Individual pairwise comparisons against Htt alone as control are shown for *p* < 0.05. *C,* death rate for cells that have formed inclusions (*i.e.* the i population). Three-way ANOVA for each chaperone treatment against Htt alone for all Gln length and time points are as follows: hsp40 (*p* = 0.004), hsp70 (*p* < 0.001), and hsp40 and hsp70 (*p* = 0.220). Individual pairwise comparisons against Htt alone as control are shown for *p* < 0.05. *D,* death rate for transfected cells lacking inclusions (ni population). Three-way ANOVA for each chaperone treatment against Htt alone for all Gln length and time points are as follows: hsp40 (*p* = <0.001), hsp70 (*p* = 0.002), and hsp40 and hsp70 (*p* < 0.001). Individual pairwise comparisons against Htt alone as control are shown for *p* < 0.05.

We next investigated the role of chaperones in mediating the toxicity of Httex1 aggregation using the pTIREX vectors and SYTOX, D, and L gating strategies. Assessment of the total population of transfected cells for extent of inclusion formation showed that, as per Fig. 3, hsp40 reduced inclusion formation on average by about 20–30% and that hsp70 had little, if any, effect (Fig. 4*A*). Compared with 25Gln control for base-line level of toxicity, each expanded polyQ length led to a higher level of toxicity that was most pronounced by 46 h of expression (up to 15% of cells above 25Gln). However, there was no noticeable benefit in viability of cells upon overexpression of hsp40 (perhaps even a small detrimental effect for some conditions) (Fig. 4*B*), whereas hsp70 had a minor but significant benefits (Fig. 4*B*). Assessment of the subpopulation enriched with inclusions (the i population) revealed a far greater level of cell death (up to 50–75% of cells at 46 h; Fig. 4*C*) than for the subpopulation lacking inclusions (15–30% at the same time point; Fig. 4*D*), consistent with inclusion formation correlating closely with cell death. Chaperone overexpression produced two notable effects on these death rates. The first was that hsp70 conferred a large reduction in death rates (about 15% at 46 h) for cells with inclusions and no noticeable difference for cells lacking inclusions (Fig. 4, *C* and *D*). hsp40, however, conferred no benefit and was possibly detrimental to cell survival with or without inclusions. The second notable effect was that coexpression of hsp40 and hsp70 increased the death rate of cells lacking inclusions by about 5–10% at 46 h (Fig. 4*D*) and removed the benefit to survival of cells expressing hsp70 alone (Fig. 4*C*). Collectively, these data point to hsp40 having a large effect on suppressing aggregation in a manner without protecting the cells from toxicity and possibly introducing additional toxicity. hsp70 in contrast had no effect on aggregation but improved the survival of cells that had formed inclusions. The hsp40·hsp70 combination more greatly reduced aggregation, but it also augmented toxicity.

**FIGURE 5.**
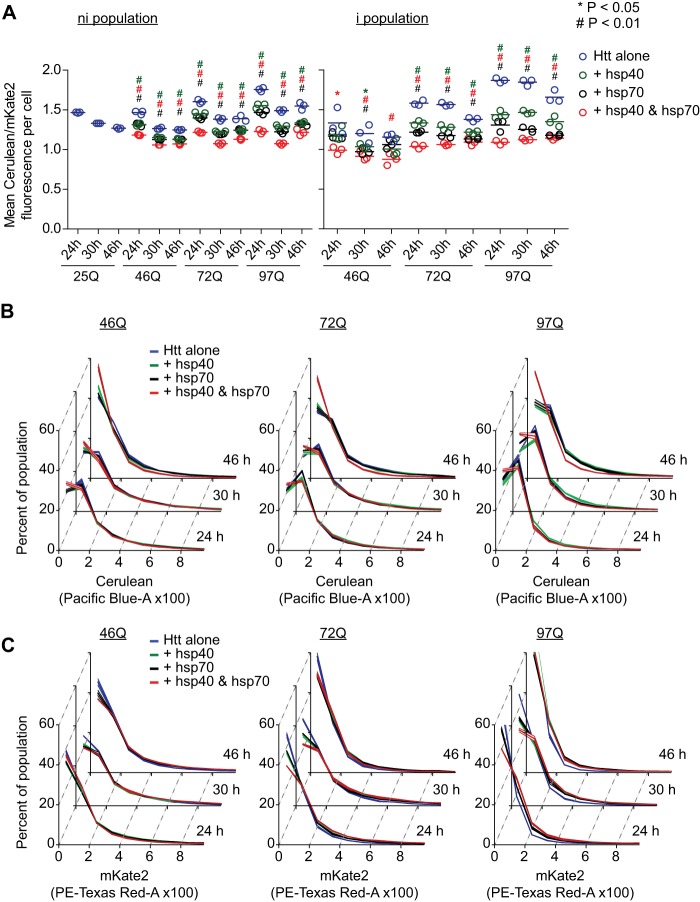
**Chaperone treatment reduces Httex1 abundance and increases reporter mKate2.** *A,* data show population means of mKate2-F/Cerulean for flow cytometry data in the M gate (see Fig. 1) subdivided into cells lacking inclusions (ni population) and those with inclusions (i population). Mean of the three replicates shown as *bars*. Treatments are as described in Fig. 3 legend. *B,* absolute abundance of Httex1-Cerulean in the cell population as assessed by Cerulean fluorescence histograms. Treatments are as described in Fig. 3 legend. Data show mean (central lines) ± S.D. (outer boundaries); *n* = 3. For ANOVA, individual pairwise comparisons against Htt alone as control are shown for *p* < 0.05. *C*, absolute abundance of mKate2 in the cell population as assessed mKate fluorescence histograms. Treatments are described in Fig. 3 legend. Data show mean (central lines) ± S.D. (outer boundaries); *n* = 3. For ANOVA, individual pairwise comparisons against Htt alone as control are shown for *p* < 0.05.

Because the hsp40·hsp70 combination reduced the overall number of cells with inclusions (compared with hsp40 alone treatment), but did not change the extent of inclusions in cells when matched for expression level (Fig. 3), this suggested that the hsp40·hsp70 treatment decreased the abundance of Httex1 in each cell (*e.g.* by promoting degradation), which would slow the aggregation process of Httex1 collectively within the population of cells. To investigate this possibility, we exploited the capacity of the IRES vector system for titrating expression levels of mKate2-F with Httex1-Cerulean to establish relative differences in Httex1-Cerulean in individual cells. By gating only live cells (SYTOX−, M cells as depicted in Fig. 1*C*), the relative levels of Htt could be related to mKate2 in each cell. Each chaperone treatment, and to the greatest extent hsp40·hsp70, decreased the level of Httex1-Cerulean relative to mKate2 consistent with the hypothesis that the chaperones enhance clearance of Httex1-Cerulean (Fig. 5*A*). This was true for cells lacking inclusions (*i.e.* the ni population) and cells with inclusions (the i population). To investigate the effect on Httex1 levels in more detail, we examined the absolute levels of Httex1 based on the Cerulean fluorescence level histograms (Fig. 5*B*) and that of mKate2 (Fig. 5*C*). The chaperone treatments shifted the population modestly to lower levels of Httex1-Cerulean expression. The effect was most pronounced with the hsp40·hsp70 and at 46 h, which supports the conclusion that supplementing the cells with hsp40·hsp70 enhances the cellular capacity to degrade Httex1 in addition to a role for hsp40 suppressing aggregation. Unexpectedly, however, was the effect of the chaperone treatments on mKate2. Each treatment, and most pronouncedly hsp40·hsp70, increased the levels of mKate2 (Fig. 5*C*). Furthermore, the relative increases correlated positively with increasing Gln length. These results suggest that the additional hsp40·hsp70 can increase the efficiency of mKate2 folding under conditions of stress conferred by pathogenic polyQ lengths of Httex1. Hence, the data points to the chaperones changing the efficiency of folding and degradation processes in *trans*, with mKate2 folding favored and at the same time Httex1 more extensively degraded.

**FIGURE 6.**
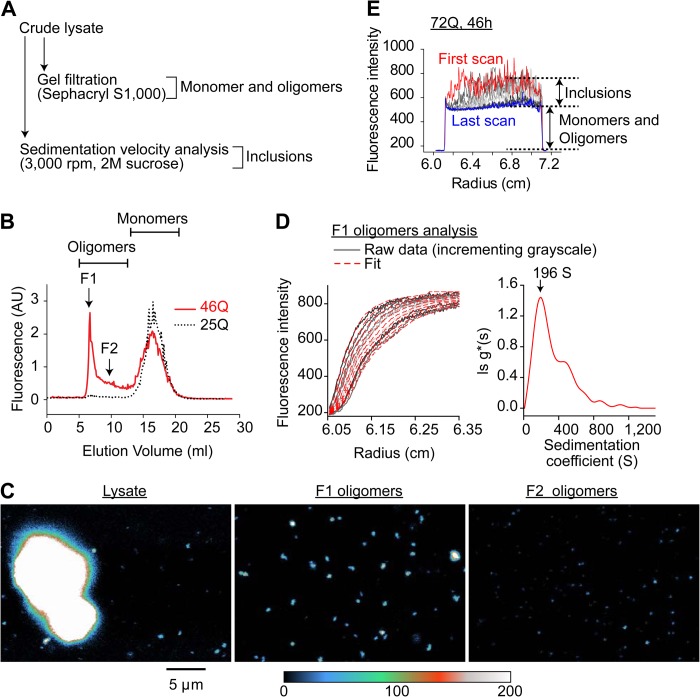
**Relating the biochemical state of Httex1 as monomers, oligomers, and inclusions in Neuro2a cell lysates with the flow cytometry workflow.** *A,* schematic of the workflow used to calculate the molecular proportions of Httex1 as monomers, oligomers, and inclusions. *B,* gel filtration chromatography with Sephacryl S1000 resin to elute Httex1-Emerald from (nondenatured) Neuro2a cell lysates. *C,* confocal microscopic images of oligomer-rich fractions F1, F2, and the crude lysate of the 46Gln form of Httex1-Emerald. *D,* fraction F1 was analyzed by sedimentation velocity analysis at a rotor speed of 3,000 rpm, 11 °C, with scans taken continuously (*grayscale*). Data were fitted to a model describing a distribution of noninteracting molecules (ls − g*(*s*)). The mode sedimentation coefficient (*s*_20,_*_w_* = 196 S) is indicated on the sedimentation coefficient distribution derived from the fit. *E,* strategy to calculate inclusion load by SVA of lysate in 2 m sucrose at 3,000 rpm. Under these conditions only inclusions sediment are indicated.

To quantitate the effect of the chaperones on the Httex1 oligomeric state at the biochemical level, we devised a strategy to quantitate Httex1-Emerald aggregate forms into three basic classes as follows: monomers, oligomers, and inclusions (summarized scheme in Fig. 6*A*). We previously showed by SVA that Httex1-Emerald fusions form only monomers in Neuro2a cells in context of the nonaggregating 25Gln form, whereas the 46Gln counterpart forms monomers (diameter of ∼5 nm), a pool of oligomers with a predominant size of 140 S (diameter of ∼30 nm), and inclusions (diameter of ∼5–10 μm) (18). Based on these physical dimensions, we hypothesized that gel filtration chromatography on Sephacryl S1000 resin should resolve the oligomers from the monomers, which has a resolving range of ∼20–300 nm for spherical particles (43). S1000 gel filtration occluded all inclusions from passing the column, whereas the oligomers came out in a broad peak separately from the monomers (Fig. 6, *B* and *C*). SVA at low speed (3,000 rpm angular velocity) confirmed that the oligomers eluting from the column were comparable in size to the pool we previously reported in whole unfractionated lysate (mode *s* = 140 S in lysate (18) *cf*. 196 S here) (Fig. 6*D*). Because the inclusions were occluded from entering the gel filtration column, which hinders a capacity to quantitate total inclusion load directly by gel filtration, we also performed SVA of crude lysate adjusted to have high viscosity (by the addition of sucrose to 2 m) at low angular speed (3,000 rpm), which we previously showed slowed the sedimentation of inclusions sufficiently to enable them to be detected by SVA (18). Under these conditions, inclusions were the only molecular forms of Httex1-Emerald that sedimented and formed a sedimenting boundary from which we could calculate the proportion of Htt molecules in an inclusion state (Fig. 6*E*).

**FIGURE 7.**
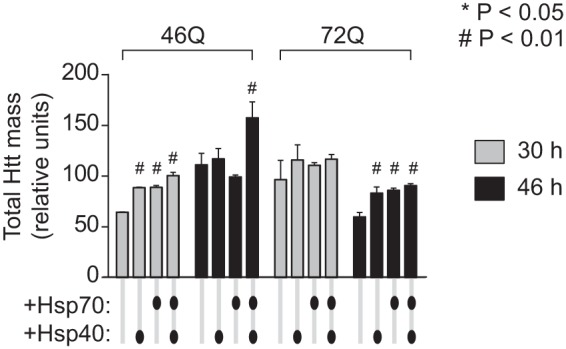
**hsp40 and hsp70 increase total abundance of Httex1 molecules in the cell population.** The total amount of Httex1-Emerald molecules (based on Emerald fluorescence intensities) in Neuro2a cell lysate is shown as defined by gel filtration (which can quantitate monomers and oligomers) and SV analysis in 2 m sucrose at 3,000 rpm (which can quantitate inclusions). Cells were transfected with equal DNA quanta of Httex1-Emerald and hsp40 and hsp70, buffered with the GFP^inv^ construct under the same conditions as for the flow cytometry data in Figs. 3 and 4. *n* = 3, mean ± S.D. shown. For ANOVA, individual pairwise comparisons against Htt alone as control are shown for *p* < 0.05.

**FIGURE 8.**
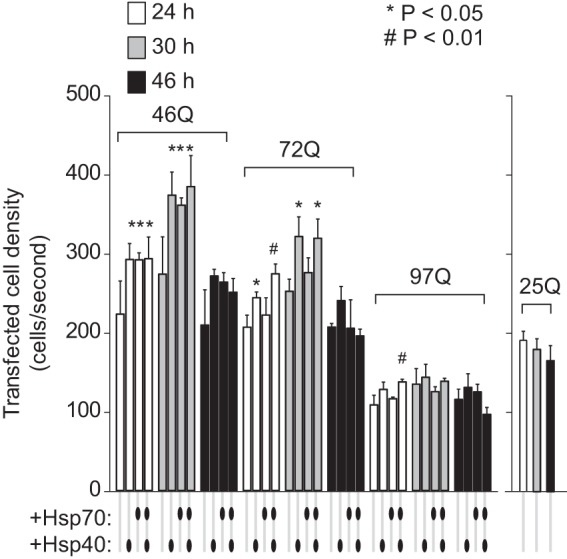
**hsp40 and hsp70 influence on cell number in population.** The relative populations of cells were estimated based on the density of cells in solution for flow cytometry analysis. This was assessed by counting the cell counts per standard flow rate for the transfected population. For 46Gln and 97Gln, *n* = 3, mean ± S.D. shown. For 25Gln, *n* = 2, mean ± S.D. shown. For ANOVA, individual pairwise comparisons against Htt alone as control are shown for *p* < 0.05.

The combined gel filtration and SVA data enabled the determination of levels of monomers and oligomers (by gel filtration) and inclusions and total abundance of Httex1-Emerald molecules (by low speed, 2 m sucrose SVA). We examined the 46Gln and 72Gln Httex1 variants at time points post-transfection that that had pronounced divergent influences from hsp40 and hsp70 (30 and 46 h) based on the data in Figs. 3 and 4. The chaperone coexpressions collectively mildly increased the total yield of Httex1-Emerald molecules, with the hsp40·hsp70 treatment conferring the greatest extent (Fig. 7). This result is seemingly inconsistent with the prior experiments showing the chaperones decrease the steady state levels of Httex1 with each cell (Fig. 5). However, if the chaperone treatments are partially rescuing defects in quality control caused by pathogenic Htt, then these results could be consistent with an overall improvement in growth rates of the cells that compensates for the reduced Httex1-Cerulean levels within individual cells. To examine this possibility, we estimated the number of cells in our culture system as a proxy of growth rate by measuring the density of transfected cells in a fixed volume after harvesting at different time points using flow cytometry count rate data (Fig. 8). Indeed, the chaperone treatments generally increased the number of cells. This effect was most notable for the 46Gln treatments relative to longer polyQ lengths, and also for earlier time points for all polyQ treatments. Because longer polyQ lengths and longer time points both positively correlate with greater stress to the cells, this result is consistent with the chaperones enabling a faster rate of growth under milder conditions of polyQ stress. Hence, the chaperones, most notably the hsp40·hsp70 treatment, seem to foster both an increased turnover of Htt in addition to a greater number of cells in the population.

**FIGURE 9.**
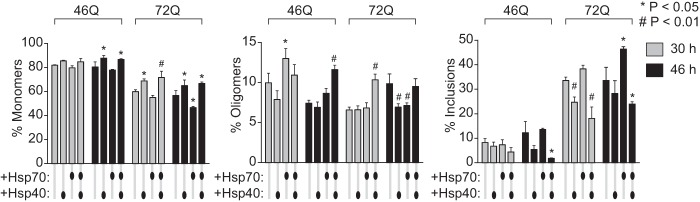
**hsp40 and hsp70 influence on molecular partitioning of Httex1 as monomers, oligomers, and inclusions.** The proportion of Httex1-Emerald molecules in Neuro2a cell lysate is shown as defined by gel filtration (which can quantitate monomers and oligomers) and SV analysis in 2 m sucrose at 3,000 rpm (which can quantitate inclusions). Cells were transfected with equal DNA quanta of Httex1-Emerald and hsp40 and hsp70, buffered with the GFP^inv^ construct under the same conditions as for the flow cytometry data in Figs. 3 and 4. Total abundance of Htt based on fluorescence yield in lysate (*n* = 3, mean ± S.D. shown). For ANOVA, individual pairwise comparisons against Htt alone as control are shown for *p* < 0.05. Proportions of monomers, oligomers, and inclusions (*n* = 3, mean ± S.D. shown). For ANOVA, individual pairwise comparisons against Htt alone as control are shown for *p* < 0.05.

The proportion of Httex1 molecules in the lysate as monomer, oligomer, and inclusion is shown in Fig. 9. hsp70 overexpression showed a trend to mildly increase the total proportion of Httex1-Emerald molecules in inclusions, most evident at 46 h, and a concordant decrease in the monomer pool. In contrast, hsp40 and slightly more effectively, the hsp40·hsp70 combination decreased Httex1 in inclusions but elevated the proportion as monomers. This result is consistent with a role for hsp40 in repressing aggregation of Httex1 and hsp70 in protecting cells with inclusions from death and the combination reducing the abundance of Httex1 within each cell.

**FIGURE 10.**
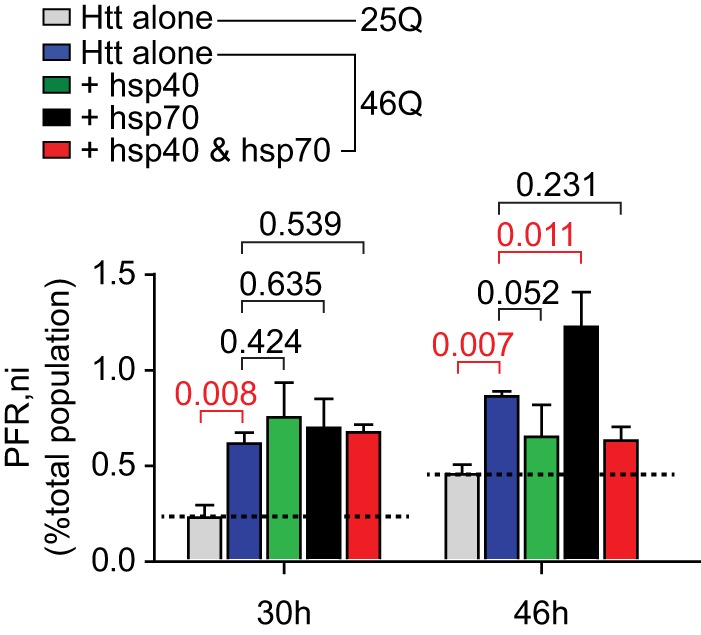
**hsp70 leads to a sustained population of cells enriched with oligomers and lacking an inclusion.** Abundance of Neuro2a cells enriched with Httex1^TC9^-Cerulean oligomers using FlAsH reactivity and gating for the PFR population and high FlAsH-reactive population is described in Fig. 2. Treatments are as described in Fig. 3 legend. Differences were assessed by one-way ANOVA using 46Gln alone as the control (*n* = 3, mean ± S.D. shown, with *p* values indicated and significant differences indicated in *red*).

A noteworthy result was for the hsp40·hsp70 to increase the oligomer pool. A similar effect was observed transiently for hsp70 alone for the 46Gln form of Httex1 (Fig. 9). One explanation for this result is that these oligomers were hsp40·hsp70 complexes engaged with Httex1 as a client, in effect as a triage for refolding or degradation (44). To probe how the oligomers form in individual cells in context with the chaperone treatment, we employed our TC9 biosensor form of Httex1 (Httex1^TC9^-Cerulean) to monitor cells enriched with oligomers but lacking an inclusion. Httex1^TC9^-Cerulean only binds FlAsH when it is in a monomeric state based on *in vitro* aggregation reactions (17). Hence, cells containing Httex1^TC9^-Cerulean that are poor FlAsH-reactive (PFR) are indicative of most Htt molecules adopting a biochemically self-aggregated state (gating strategy shown in Fig. 2*E*) (19). Cells that are PFR and that lack an inclusion (PFR,ni) suggest an intermediate stage whereby the dispersed Htt monomers have spontaneously self-aggregated (19). The 46Gln form of Htt^TC9^-Cerulean revealed ∼0.5% of the total transfected population to be enriched with oligomers (*i.e.* PFR,ni) (Fig 10). At 30 h, there was no significant difference between the chaperone treatments for this population. However, by 46 h, hsp70 led to a sustained, significant increase of this population, whereas the hsp40 and hsp40·hsp70 treatments led to a mild decrease (but which was not significant). These data suggest oligomer-enriched cells do not seem to correlate with the increased abundance of oligomers at the molecular level (at least for the hsp40·hsp70 treatments) supporting the conclusion that the oligomers at the biochemical level in context of the hsp40·hsp70 treatments are of a different molecular nature to self-aggregated Httex1. Hence, the hsp70 treatment showing a small number of additional cells enriched with oligomers may reflect hsp70 overall providing a survival benefit to cells undergoing Httex1 aggregation.

## DISCUSSION

Our study brings together a number of new approaches that enable the aggregation process of mutant Httex1 in a cell to be related to cell death. The findings can be summarized as follows. Overexpression of hsp40 suppressed aggregation of Httex1 into inclusions and stabilized Httex1 as monomers. This effect was not beneficial to cell viability. hsp70 did not alter the aggregation of Httex1 in inclusions but protected the cells with inclusions from death. When added together, hsp40 and hsp70 enhanced Httex1 turnover and seemed to partially rescue Httex1-mediated defects in the folding efficiency of the reporter protein mKate2 and improved cellular proliferation. Collectively, these data paint a picture of pathogenic Httex1 severely stressing the quality control network, which in turn negatively impacts on general housekeeping functions, a process that can be partially restored by the supplementation of additional hsp40 and hsp70.

One paradoxical conclusion was the correlation of greater toxicity to cells when treated with both hsp40 and hsp70. This result may reflect an incomplete supplementation of dysfunctional protein quality control caused by Httex1 in a manner that confers a dominant negative phenotype. This could arise by hsp40 and hsp70 forming canonical complexes with substrates in response to the stressed cell that becomes stalled in delivering substrates to downstream quality control processes due to a lack of additional cofactors that are redirected to managing the overwhelming Httex1 toxicity. Recent data suggest that polyQ aggregation can selectively sequester essential elements of protein quality control machinery (including DNAJB proteins) that prevent proper operation of quality control networks, which is consistent with this type of mechanism (45). In addition, the overexpression of both hsp40 and hsp70 together increased the total pool of oligomers that have features consistent with canonical hsp40·hsp70·Htt client complexes that have been observed previously for Httex1 (44).

Hence, a likely possibility for the beneficial effect of hsp70 is that it partly compensates for a loss of cellular quality control capacity that has been redirected to managing Httex1 aggregation/dysfunction. An increasing body of evidence suggests protein aggregation correlates with a collapse of quality control systems (45–47). Yet, it seems that aggregation itself may not be the driver of the collapsed quality control systems and rather soluble forms of Httex1 impart cell stress, perhaps by their intrinsic proneness to aggregation imparting a capacity to interfere with other cellular functions. Prior work has pointed to soluble forms of Httex1 correlating more closely to cell death and dysfunction than aggregated states that support this mechanism (11, 48–52). PolyQ has been previously suggested to adopt heterogeneous disordered monomer conformations of unusual mechanical rigidity and compaction as a result of intrachain glutamine side chain hydrogen bonding (53–58). It is tempting to suggest that compact hairpin monomers are directly pernicious to the cell or indirectly are toxic because of an extreme tendency to aggregate. Our results also confer more generally with findings that hsp70 (at least the isoform HSPA1A) does not reduce aggregation but still provides protection from polyQ-mediated toxicity upon aggregation (18, 30–33, 35, 42). Other studies have also suggested that hsp70 can remodel Httex1 into benign aggregate structures, suggesting a possible role for hsp70 in altering the type of aggregates that are present (29, 44).

In summary, our methodological platform offers a new capacity to view the impact of aggregation kinetics in the cell. This platform enables expression level dependence to be tracked at a finer grain of resolution than previously possible and has potential for further adaptation in multicolor fluorescence imaging for tracking how each cell type correlates with cellular functionality and health. A notable feature of our platform is its ability to estimate cell death in the entire culture over a typical plate-based imaging platform that requires cells to remain adherent. This offers benefits for investigation of phenomena relating to dysfunction and toxicity and tracking of all cells that may have detached through cytotoxicity. This strategy should be readily adaptable to other systems and especially for proteins that already have employed TC tag approaches to label different conformations of proteins at the molecular level (38, 59–61).

## Footnotes

2 The abbreviations used are:

Httex1: Htt exon 1
polyQ: polyglutamine
TC: tetracysteine
PulSA: pulse shape analysis
SVA: sedimentation velocity analysis
ANOVA: analysis of variance
PFR: poor FlAsH-reactive
IRES: internal ribosome entry site.
